# Supplementary material for: Multi‐dataset identification of innovative feature genes and molecular mechanisms in keratoconus
Source: J Cell Mol Med. 2024 Sep 19;28(18):e70079. doi: 10.1111/jcmm.70079 (PMC11412914; doi:10.1111/jcmm.70079)
Supplement: Supplementary file 2 — Material S2. [file JCMM-28-e70079-s002.zip › 7.GSEA analysis/6.SNORA74B/KEGG_RIBOSOME.html]

Details for gene set KEGG\_RIBOSOME[GSEA]

|  || Dataset | SNORA74B.group.cls#h\_versus\_l |
| Phenotype | group.cls#h\_versus\_l |
| Upregulated in class | h |
| GeneSet | KEGG\_RIBOSOME |
| Enrichment Score (ES) | 0.2895496 |
| Normalized Enrichment Score (NES) | 1.0738534 |
| Nominal p-value | 0.33130082 |
| FDR q-value | 1.0 |
| FWER p-Value | 0.996 |
Table: GSEA Results Summary

  

Fig 1: Enrichment plot: KEGG\_RIBOSOME      
 Profile of the Running ES Score & Positions of GeneSet Members on the Rank Ordered List

  

| SYMBOL | TITLE | RANK IN GENE LIST | RANK METRIC SCORE | RUNNING ES | CORE ENRICHMENT || 1 | RPL3L | na | 8409 | 0.127 | -0.1887 | Yes |
| 2 | RPL32 | na | 8780 | 0.119 | -0.1488 | Yes |
| 3 | RPL27A | na | 9795 | 0.097 | -0.1368 | Yes |
| 4 | RPS4Y1 | na | 11315 | 0.070 | -0.1510 | Yes |
| 5 | RPS4X | na | 13804 | 0.038 | -0.2065 | Yes |
| 6 | RPS6 | na | 15324 | 0.021 | -0.2412 | Yes |
| 7 | RPS9 | na | 15775 | 0.017 | -0.2470 | Yes |
| 8 | RPS27 | na | 16249 | 0.012 | -0.2555 | Yes |
| 9 | RPL36AL | na | 16305 | 0.011 | -0.2522 | Yes |
| 10 | RPL36 | na | 16389 | 0.011 | -0.2501 | Yes |
| 11 | RPS20 | na | 16549 | 0.009 | -0.2509 | Yes |
| 12 | RPL36A | na | 16644 | 0.008 | -0.2502 | Yes |
| 13 | RPL39 | na | 16802 | 0.006 | -0.2520 | Yes |
| 14 | RPL23 | na | 17123 | 0.004 | -0.2597 | Yes |
| 15 | RPL12 | na | 17298 | 0.002 | -0.2641 | Yes |
| 16 | RPLP2 | na | 17629 | -0.002 | -0.2728 | Yes |
| 17 | RPL8 | na | 17665 | -0.002 | -0.2728 | Yes |
| 18 | RPL26 | na | 17700 | -0.003 | -0.2727 | Yes |
| 19 | RPL38 | na | 17733 | -0.003 | -0.2723 | Yes |
| 20 | FAU | na | 17790 | -0.004 | -0.2725 | Yes |
| 21 | RPS12 | na | 17938 | -0.005 | -0.2744 | Yes |
| 22 | RPS23 | na | 17994 | -0.006 | -0.2735 | Yes |
| 23 | RPL14 | na | 18250 | -0.009 | -0.2771 | Yes |
| 24 | RPL31 | na | 18283 | -0.009 | -0.2741 | Yes |
| 25 | RPL6 | na | 18338 | -0.010 | -0.2714 | Yes |
| 26 | RPL11 | na | 18363 | -0.010 | -0.2677 | Yes |
| 27 | RPS28 | na | 18464 | -0.011 | -0.2657 | Yes |
| 28 | RPL24 | na | 18492 | -0.012 | -0.2615 | Yes |
| 29 | RPS18 | na | 18675 | -0.014 | -0.2609 | Yes |
| 30 | RPL4 | na | 18769 | -0.015 | -0.2574 | Yes |
| 31 | RPS15 | na | 18885 | -0.016 | -0.2540 | Yes |
| 32 | RPS7 | na | 18902 | -0.016 | -0.2477 | Yes |
| 33 | RPL35 | na | 18934 | -0.016 | -0.2416 | Yes |
| 34 | RPL34 | na | 18954 | -0.016 | -0.2351 | Yes |
| 35 | RPS15A | na | 18974 | -0.017 | -0.2286 | Yes |
| 36 | RPL13 | na | 19004 | -0.017 | -0.2222 | Yes |
| 37 | RPS8 | na | 19023 | -0.017 | -0.2153 | Yes |
| 38 | RPL27 | na | 19034 | -0.017 | -0.2082 | Yes |
| 39 | RPS25 | na | 19036 | -0.017 | -0.2008 | Yes |
| 40 | MRPL13 | na | 19041 | -0.017 | -0.1934 | Yes |
| 41 | RPL26L1 | na | 19130 | -0.019 | -0.1881 | Yes |
| 42 | RPS29 | na | 19200 | -0.020 | -0.1817 | Yes |
| 43 | RPS13 | na | 19240 | -0.020 | -0.1742 | Yes |
| 44 | RPL19 | na | 19248 | -0.020 | -0.1658 | Yes |
| 45 | RPS3 | na | 19365 | -0.021 | -0.1600 | Yes |
| 46 | RPL10L | na | 19398 | -0.022 | -0.1516 | Yes |
| 47 | RPL41 | na | 19415 | -0.022 | -0.1426 | Yes |
| 48 | RPS2 | na | 19595 | -0.024 | -0.1375 | Yes |
| 49 | RPS5 | na | 19669 | -0.025 | -0.1290 | Yes |
| 50 | RPL17 | na | 19678 | -0.025 | -0.1186 | Yes |
| 51 | RPL37A | na | 19733 | -0.026 | -0.1092 | Yes |
| 52 | RPS24 | na | 19736 | -0.026 | -0.0983 | Yes |
| 53 | RPL35A | na | 19827 | -0.027 | -0.0896 | Yes |
| 54 | UBA52 | na | 19838 | -0.027 | -0.0785 | Yes |
| 55 | RPL7A | na | 19897 | -0.027 | -0.0685 | Yes |
| 56 | RPL21 | na | 19967 | -0.028 | -0.0586 | Yes |
| 57 | RPL18 | na | 19972 | -0.028 | -0.0467 | Yes |
| 58 | RPL23A | na | 19987 | -0.028 | -0.0351 | Yes |
| 59 | RPS10 | na | 19995 | -0.028 | -0.0232 | Yes |
| 60 | RPS16 | na | 20065 | -0.029 | -0.0128 | Yes |
| 61 | RPS21 | na | 20074 | -0.029 | -0.0006 | Yes |
| 62 | RPL29 | na | 20102 | -0.029 | 0.0112 | Yes |
| 63 | RPL13A | na | 20128 | -0.030 | 0.0231 | Yes |
| 64 | RPLP1 | na | 20137 | -0.030 | 0.0356 | Yes |
| 65 | RPL30 | na | 20161 | -0.030 | 0.0477 | Yes |
| 66 | RPS11 | na | 20199 | -0.031 | 0.0597 | Yes |
| 67 | RPL10A | na | 20258 | -0.031 | 0.0713 | Yes |
| 68 | RPLP0 | na | 20267 | -0.031 | 0.0844 | Yes |
| 69 | RPL22 | na | 20330 | -0.032 | 0.0962 | Yes |
| 70 | RPL22L1 | na | 20366 | -0.032 | 0.1090 | Yes |
| 71 | RPL7 | na | 20372 | -0.032 | 0.1227 | Yes |
| 72 | RPL5 | na | 20393 | -0.033 | 0.1360 | Yes |
| 73 | RPS19 | na | 20482 | -0.034 | 0.1480 | Yes |
| 74 | RPL18A | na | 20754 | -0.037 | 0.1560 | Yes |
| 75 | RPS17 | na | 20838 | -0.038 | 0.1698 | Yes |
| 76 | RPL15 | na | 21046 | -0.041 | 0.1811 | Yes |
| 77 | RPL9 | na | 21171 | -0.042 | 0.1955 | Yes |
| 78 | RPS3A | na | 21249 | -0.043 | 0.2115 | Yes |
| 79 | RPS27A | na | 21294 | -0.043 | 0.2288 | Yes |
| 80 | RPS26 | na | 21804 | -0.049 | 0.2352 | Yes |
| 81 | RPL3 | na | 21920 | -0.051 | 0.2536 | Yes |
| 82 | RPL37 | na | 22008 | -0.052 | 0.2732 | Yes |
| 83 | RPS27L | na | 22347 | -0.056 | 0.2874 | Yes |
| 84 | RSL24D1 | na | 23307 | -0.070 | 0.2895 | Yes |
| 85 | RPL28 | na | 24756 | -0.094 | 0.2878 | No |
Table: GSEA details [plain text format]

  

Fig 2: KEGG\_RIBOSOME      
 Blue-Pink O' Gram in the Space of the Analyzed GeneSet

  

Fig 3: KEGG\_RIBOSOME: Random ES distribution      
 Gene set null distribution of ES for **KEGG\_RIBOSOME**
